# Doctors, Midwives, and Patients

**Published:** 1902-03-29

**Authors:** 


### DOCTORS, MIDWIVES, AND PATIENTS.

Amid the various discussions which have taken place in regard to the registration of the midwives, we have perhaps to some extent lost sight of what will in practice be fundamental questions, namely, What are to be the duties of the midwife? and what her relation to qualified medical practitioners? It has been generally taken for granted that the work of the midwife will be attendance upon ordinary cases, and that it will be her duty to send for a doctor if anything abnormal turns up. But as a fact the early diagnosis of abnormalities often demands a considerable degree of skill, greater perhaps than might be required for the treatment of the abnormalities discovered. Hence any conception of the proper relations between the doctor and the midwife which hinges on the assumption that the midwife will send for help when things go wrong, involves the further assumption that the midwife is a much more skilful and a much more humble minded person than she will probably turn out to be; and the question should be seriously faced whether this idea of a midwife's duties, namely, that she is to attend all sorts of cases in the first instance, and send for the doctor if anything should go wrong, is a proper or even a justifiable one, and whether some serious and systematic endeavour should not be made to ascertain beforehand in which cases accidents are likely to happen, and thus to divide the cases, from the very beginning, into two classes—those which may be safely left to midwives and those which ought to be retained under the care of fully qualified practitioners.

In a lecture delivered before the London Hospital Medical Society, Dr. Herman [1] very forcibly points out the evils which must result unless some such plan be adopted. He says that it is common in poor neighbourhoods for midwives to co-operate with medical men, and that as a rule such co-operation is carried on in the worst possible way. If there is hæmorrhage the midwife will send for the doctor but if there is a head presentation she waits and watches; if the patient gets into a dangerous state then she sends for the doctor. The patient is allowed to drift into danger, and when the doctor is sent for it is generally too late for him to be of use. This is not the best use to make of the doctor, for he cannot do the best thing for the patient unless he sees the case early. Dr. Herman suggests that a doctor who practises in a neighbourhood such that he is obliged to attend a number of poor women, should himself employ midwives, and that when poor women, who cannot afford his fee for acting as an anæsthetist, come to ask his attendance in child-birth he should explain to them the far greater benefit of preventing difficulty than of treating it, and should insist on the patient coming to him for examination when she is eight months pregnant, and again when she has reached the full term. If at the latter period he is sure that the child is not of excessive size, that the pelvis is normal, and that the head is engaged in it, then he should explain to the patient that she will in all probability be delivered naturally, and that the midwife can do all that is required after the child is born; that the patient should send for him as soon as she is taken in labour, in order that he may ascertain that everything is still normal (for the child may have changed its position), but that if it is going to be a normal delivery she is not to expect him to sit by and watch it. He very properly asks, What is there for a doctor to do in a case of normal labour? The patient may wish for anæsthesia, but acting as an anæsthetist is not an obstetrical service, although the obstetrician may do it. Obstetrically all that the doctor has to do in natural labour is to watch the parts dilate and the child emerge, and then to tie and divide the cord, and, some half-hour after, to press the placenta out of the vagina. "Does it need a highly educated man to do this? I think that the average woman could do these simple things as well as the most highly-trained doctor, and I think it a pure waste of time for a doctor to sit by watching the passage dilate in a case of normal labour." With all due respect to Dr. Herman we must remark that it is not for the sake of doing this that the doctor does attend cases of normal labour, but for the sake of being at hand in case any emergency should arise, and that the possibility of adopting Dr. Herman's suggestion as to the mode in which co-operation between doctors and midwives should be carried out depends almost entirely upon the degree to which it is possible by early examination to eliminate from the crowd of normal cases the comparatively small percentage in which abnormalities exist, and further upon the extent to which accidents, such as post-partum hæmorrhage, are confined to the cases which can be so set apart.

[1] Clinical Jour., Mar. 5.

